# Epstein-Barr virus positive diffuse large B-cell lymphoma predict poor outcome, regardless of the age

**DOI:** 10.1038/srep12168

**Published:** 2015-07-23

**Authors:** Ting-Xun Lu, Jin-Hua Liang, Yi Miao, Lei Fan, Li Wang, Xiao-Yan Qu, Lei Cao, Qi-Xing Gong, Zhen Wang, Zhi-Hong Zhang, Wei Xu, Jian-Yong Li

**Affiliations:** 1 Department of Hematology, the First Affiliated Hospital of Nanjing Medical University, Jiangsu Province Hospital, Nanjing 210029, China; 2 Department of Pathology, the First Affiliated Hospital of Nanjing Medical University, Jiangsu Province Hospital, Nanjing 210029, China; 3 Collaborative Innovation Center for Cancer Personalized Medicine, Nanjing Medical University, Nanjing 210029, China

## Abstract

Epstein-Barr virus (EBV) positive diffuse large B-cell lymphoma (DLBCL) of the elderly is defined as patients older than 50 years alone. However, recent studies showed young patients with sound immune status could also be affected. In this study, we investigated the clinical features and outcomes of patients with EBV positive DLBCL in the different age groups using different EBER cut-off values. The prevalence of EBV positive DLBCL was 14.0% (35/250) and 10.4% (26/250) for EBER cut-off of 20% and 50%, respectively. With both EBER cut-off values, patients with EBV DLBCL shared many unfavorable prognostic characteristics, regardless of age. EBV positive patients, both in the elderly and young groups, showed significantly worse overall survival and progression-free survival than negative cases. Moreover, no significant differences of outcomes were identified between different age groups with EBV positive DLBCL. In conclusion, EBV positive DLBCL patients, regardless of age, shared similar poor prognostic features and showed worse outcome than negative cases. We suggest that the age criterion of EBV positive DLBCL of the elderly, and possibly the name itself, be modified in future.

Diffuse large B-cell lymphoma (DLBCL) is the most common subtype of malignant lymphoma. DLBCL harboring Epstein-Barr virus (EBV) positive monoclonal B-cell proliferation in patients older than 50 years without any known immunodeficiency or prior lymphoma is termed EBV positive DLBCL of the elderly^1^,^2^. The EBV positive DLBCL of the elderly accounts for 8.7%–11.4% of all DLBCL in Asian countries^3^,^4^,^5^,^6^, but less than 5% in western nations^7^,^8^. Since the introduction of rituximab, R-CHOP has become the standard treatment for CD20 positive DLBCL^9^,^10^. The outcome of DLBCL patients is improved with R-CHOP, but the impact on the prognosis of EBV positive DLBCL patients remains controversial^5^,^6^,^11^,^12^.

Most of studies showed the outcome of elderly patients with EBV positive DLBCL treated with R-CHOP was worse than negative ones^4^,^5^,^12^,^13^,^14^,^15^,^16^. While few reports showed the impact of EBV positivity was overcome with R-CHOP especially patients received more than three cycles of therapies^7^,^11^.

Of note, recent reports demonstrated that EBV positive DLBCL could also affect younger patients (<50 years), who also showed poor response to traditional immunochemotherapy^4^,^16^,^17^,^18^,^19^. Hong *et al.*^14^ showed the prevalence of EBV positivity in the young group was less frequent (young vs. elderly: 6.7% vs. 9.3%), compared with the elderly group. However, they found the EBV positivity in the young group was not closely associated with unfavorable clinical features which was restricted to the elderly group. In addition, the poor prognostic impact of EBV positivity on overall survival (OS) and progression-free survival (PFS) was not observed in the young group, but in the elderly group alone^14^.

However, almost all of the previous studies, to our knowledge, analyzed the poor prognostic impact in the elderly or young group alone. None of them compared the clinical characteristics and prognosis between the elderly and young groups. Whether EBV positive DLBCL should be divided into two clinically distinct disease entities is still unknown. In present study, we investigated the clinical features of patients with EBV positive DLBCL and the outcome in different age groups.

## Material and methods

### Ethics statement

All patients provided informed consent in accordance with requirements of the Declaration of Helsinki, and the research project was approved by the University and Institutional Review Boards.

### Patients

According to the 2008 World Health Organization (WHO) classification, we reviewed the medical records of 250 patients who diagnosed as *de novo* DLBCL at our hospital between July 2006 and December 2014. Patients with unknown EBV status, primary central nervous system lymphoma, post-transplant lymphoproliferative disorders, primary mediastinal B-cell lymphoma, and HIV-positive DLBCL were excluded from the study. All of the patients were treated with rituximab plus chemotherapy or chemotherapy alone.

### Epstein-Barr virus-encoded RNA (EBER) *in situ* hybridization

EBER *in situ* hybridization was carried out using a fluorescein-conjugated EBER oligonucleotide probe and the purified IgG fraction of a mouse monoclonal anti-fluorescein antibody. Both 20% and 50% were applied as cut-off values for EBER positive tumour cells to assess the differences in clinical parameters, pathological features and survival differences^20^.

### Immunohistochemistry (IHC)

Antibodies applied in the study, according to the manufacturer’s instructions, included CD5 (clone EP2952, Abcam, cut-off: 30%), CD10 (clone 56C6, Dako, cut-off: 30%), CD30 (clone CON6D/B5, Abcam, cut-off: 30%), Ki-67 (clone Mib-1, Dako), Myc (clone Y69, Abcam, cut-off: 40%), Bcl2 (clone 124, Dako, cut-off: 50%), Bcl6 (clone LN22, Dako, cut-off: 30%), MUM1 (clone MUM1p, Dako, cut-off: 30%), FOXP1 (clone JC12, Abcam, cut-off: 60%), GCET1 (clone RAM341; Abcam, cut-off: 60%) and LMO2 (clone 1A9-1, Santa Cruz, cut-off: 30%). The cell of origin (COO) was classified according to Hans, Choi, Tally and Visco-Young algorithms. The specific cut-off of each antibody used in different algorithms was described previously^21^,^22^,^23^,^24^.

### Fluorescence *in situ* hybridization (FISH)

FISH analysis was performed according to the manufacturer’s instructions with MYC dual-color, break-apart translocation probe (Vysis LSI) and IGH/BCL2 dual-color, dual-fusion translocation probe (Vysis LSI). The cut-off levels for the probes were established by evaluating the split signal distribution in samples of reactive lymphoid tissues, calculating the mean number of split signals plus three times the standard deviation. The cut-off levels were 14% and 5% for MYC break apart probe and IGH/BCL2 dual-color, dual-fusion translocation probe, respectively.

### Statistical analyses

Statistical analyses were performed using SPSS software, version 20.0. Chi-square and Fisher exact tests were used to compare categorical variables . OS and PFS were defined according to Cheson 2014^25^. Survival curves were plotted by using Kaplan-Meier method and were compared by using log-rank test. For all the tests, a probability value of less than 0.05 (2-sided) was considered statistically significant.

## Results

### Prevalence of EBV positive DLBCL in the cohort

A total of 250 cases with DLBCL were included in the analysis as the whole cohort. Using 20% as cut-off, 14.0% (35/250) cases showed EBER positivity. The prevalence of EBER positivity were 15.1% (25/166) and 11.9% (10/84) in the elderly and young group, respectively. No significant difference of incidence was observed between the two groups (*P* = 0.497). When a cut-off of 50% was used for EBER positivity, the incidence of EBER positive cases was 10.4% (26/250). Accordingly, 11.4% (19/166) and 8.3% (7/84) were positive for EBER in the elderly and young groups, respectively. No significant difference of prevalence was observed between the two groups (*P* = 0.466).

### EBV positivity and clinical features

In the whole cohort, compared with EBER negative cases, EBER positive (for both cut-off values) patients showed male predominance, advanced clinical stages (stage III/IV), poor performance status (ECOG PS status 2–4) and lower response to first-line treatment. (Table 1,2). Using 20% as cut-off, EBV positivity was significantly associated with male sex (76.0% vs. 53.9%), poor PS status (40% vs. 12.5%) and lower response to first-line treatment (64.0% vs. 84.4%), compared with negative cases. In the young group, EBER positivity was strongly associated with poor PS status (30.0% vs. 5.8%) and lower response to first-line treatment (70.0% vs. 94.6%), With regard to other clinical features, although not statistically significant, EBV positive patients more frequently showed unfavorable characteristics compared with negative cases in both elderly and young groups (Table 1). However, no significant differences of these clinical characteristics were observed between different age groups. When we analyzed above parameters with a cut-off value of 50% for EBER, although fewer factors with significant differences were observed, EBV positive cases still showed majority of unfavaorable clinical features compared with negative ones, regardless of ages (Table 2).

### EBV positivity and clinical prognostic indicators

In the whole cohort, EBER positivity (for both cut-off values) was significantly associated with elevated serum C reactive protein (CRP) level, β2 microglobulin (β2M) level, CA125 level, erythrocyte sedimentation rate (ESR) and Ferritin level (Table 3,4). Using 20% as cut-off, in the elderly patients, compared with EBER negative cases, EBER positive ones more frequently had elevated serum CRP level (71.4% vs. 45.2%), β2M level (71.4% vs. 45.8%), CA125 level (61.5% vs. 29.3%), ESR (92.9% vs. 22.7%) and Ferritin level (55.6% vs. 29.3%). In the young group, EBER positivity was associated with all of above clinical prognositic factors (Table 3). Additionally, no significant differences of these prognostic features were observed between different age groups. When we analyzed those parameters with the 50% for cut-off of EBER, similar results were observed (Table 4).

### EBV positivity and pathological characteristics

In the whole cohort, EBER positivity (for both cut-off values) was significantly associated with less frequent Bcl6 expression, more common CD30 expression, higher Ki-67 expression (≥70%) (*P *< 0.001), and higher incidence of double hit lymphoma (DHL) (Table 5,6). Using 20% as cut-off, EBER positivity was associated with less frequent Bcl2 (40.0% vs. 62.1%), Bcl6 (28% vs. 65%) and FOXP1 expression (45.5% vs. 68.5%), more common CD30 (28.0% vs. 6.4%), higher Ki-67 expression (70.8% vs. 34.0%) and Myc expression (60.9% vs. 37.6%), more incidence of double protein expression (DPE) (44.0% vs. 24.8%), MYC gene rearrangement (34.4% vs. 10.7%) and double hit lymphoma (DHL) (13.0% vs. 1.0%). Other factors, including: lower CD10 expression (16.0% vs. 24.8%) and more common of non-GCB subtype with Choi (72.7% vs. 57.9%) and Visco-Young (75.0% vs. 59.3%) algorithms although not statistically significant, were shared by EBV positive patients, compared with negative cases (Table 5). In the young group, EBER positivity was associated with lower CD10 (0% vs. 28.0%) and LMO2 expression (60.0% vs. 86.1%), more common with CD30 expression (30.0% vs. 6.8%), MYC gene rearrangement (33.3% vs. 10.8%) and BCL2/IGH translocation (33.3% vs. 8.1%) (Table 5). In addition, no significantly differences were recognized among all of the pathological features between different age groups. When we analyzed those parameters with the cut-off value of 50%, similar results were observed in both age groups (Table 6).

### Survival analysis

#### Prognosis of EBV status

**Figure 1:**
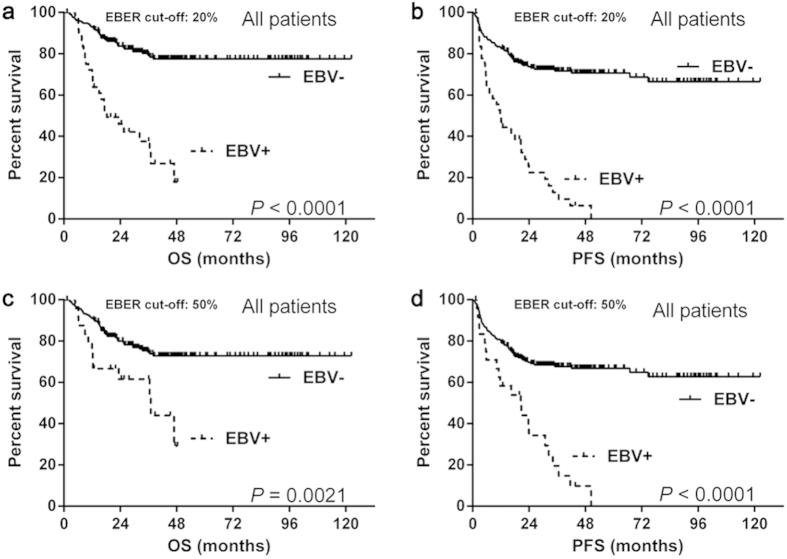
The overall survival and progession-free survival of the whole cohort. The EBV positive patients had significantly worse OS (**a**,**c**) and PFS (**b**,**d**) than the negative ones with both EBER cut-off values. Abbreviations: EBER: Epstein-Barr virus-encoded RNA; EBV: Epstein-Barr virus; OS: overall survival; PFS: progression-free survival.

In the whole cohort, after a median follow-up of 29.3 (range, 1.3–122.4) months, patients with EBV positive DLBCL showed significantly worse OS (median OS: 20% as EBER cut-off: 18.3 months vs. not reached, *P *< 0.0001; 50% as EBER cut-off: 37.0 months vs. not reached, *P* = 0.0021) (Fig. 1a,c) and PFS (median PFS: 20% as EBER cut-off: 11.9 months vs. not reached, *P *< 0.0001; 50% as EBER cut-off: 20.5 months vs. not reached, *P *< 0.0001) (Fig. 1b,d) than EBV negative ones.

#### Prognosis of EBV status in the elderly group

**Figure 2:**
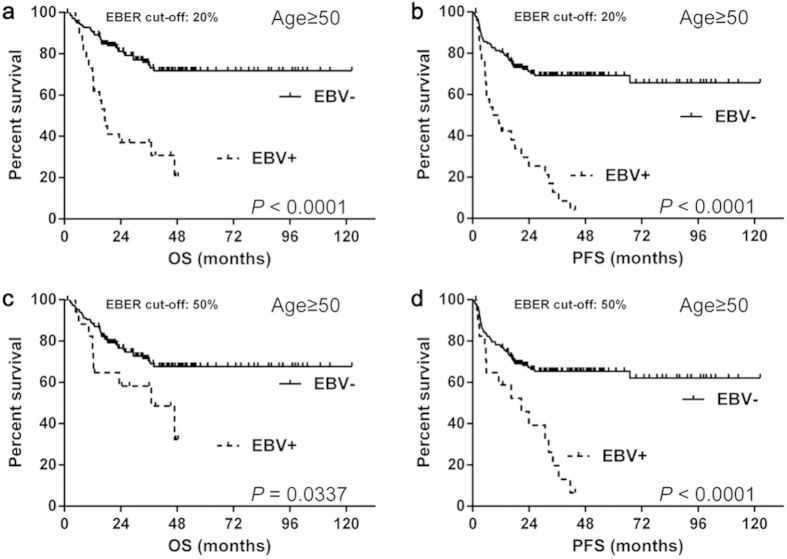
The survival differences of elderly DLBCL with EBV status. The EBV positive patients of the elderly group showed significantly worse OS (**a**,**c**) and PFS (**b**,**d**) than negative ones, regardless of the EBER cut-off values. Abbreviations: EBER: Epstein-Barr virus-encoded RNA; EBV: Epstein-Barr virus; OS: overall survival; PFS: progression-free survival.

We carried out survival analysis in the elderly group. Patients with EBV positive DLBCL showed significantly worse OS (median OS: 20% as EBER cut-off: 17.0 months vs. not reached, *P* < 0.0001; 50% as EBER cut-off: 37.0 months vs. not reached, *P* = 0.0337) (Fig. 2a,c) and PFS (median PFS: 20% as EBER cut-off: 9.8 months vs. not reached, *P* < 0.0001; 50% as EBER cut-off: 20.7 months vs. not reached, *P *< 0.0001) (Fig. 2b,d) compared with those with EBV negative DLBCL.

#### Prognosis of EBV status in the young group

**Figure 3:**
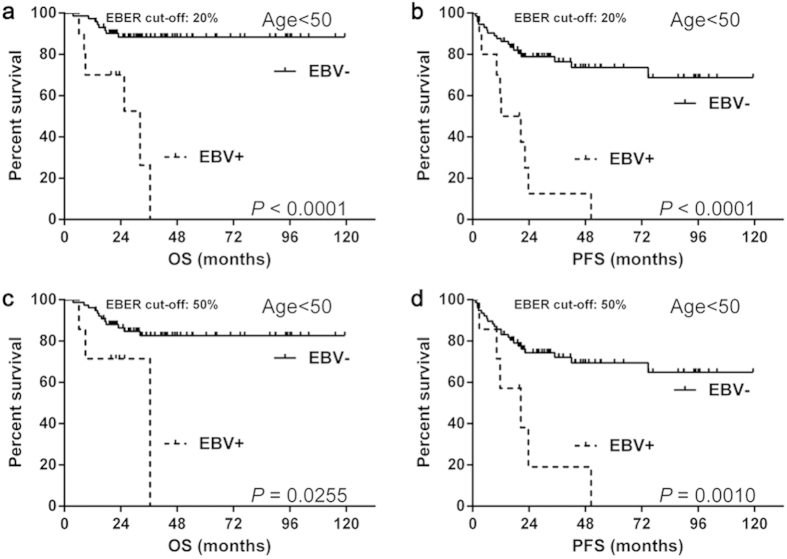
The survival differences of young DLBCL with EBV status. The EBV positive patients of the young group showed significantly worse OS (**a**,**c**) and PFS (**b**,**d**) than negative ones, regardless of the EBER cut-off values. Abbreviations: EBER: Epstein-Barr virus-encoded RNA; EBV: Epstein-Barr virus; OS: overall survival; PFS: progression-free survival.

We carried out survival analysis in the young group. Patients with EBV positive DLBCL showed significantly worse OS (median OS: 20% as EBER cut-off: 32.2 months vs. not reached, *P* < 0.0001; 50% as EBER cut-off: 36.5 months vs. not reached, *P *= 0.0255) (Fig. 3a,c) and PFS (median PFS: 20% as EBER cut-off: 16.5 months vs. not reached, *P *< 0.0001; 50% as EBER cut-off: 20.5 months vs. not reached, *P *= 0.0010) (Fig. 3c,d) than patients with EBV negative DLBCL.

#### Prognosis of EBV positivity between different age groups

**Figure 4:**
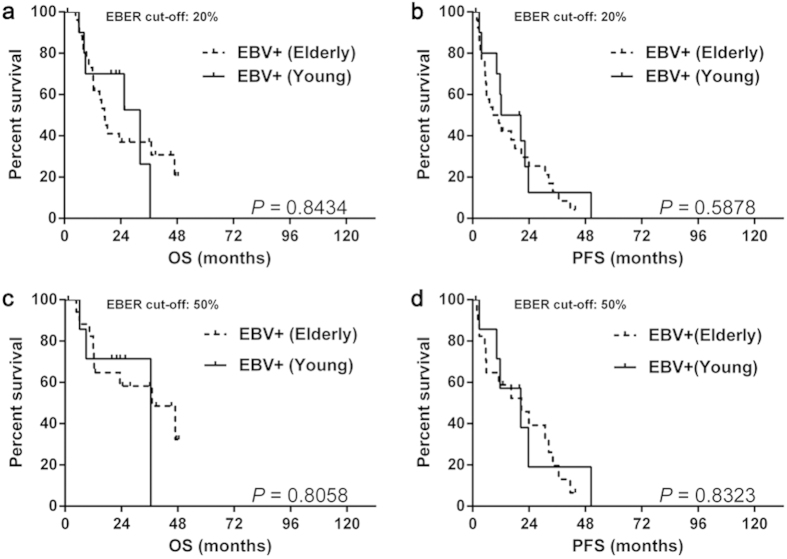
The survival differences of EBV positive DLBCL beween different age groups. The EBV positive DLBCL of the elderly group showed similar OS (**a**,**c**) and PFS (**b**,**d**) with the young ones, regardless of the EBER cut-off values. Abbreviations: EBER: Epstein-Barr virus-encoded RNA; EBV: Epstein-Barr virus; OS: overall survival; PFS: progression-free survival.

We further compared the survival difference between the elderly and young group. Unexpectedly, elderly patients with EBV positive DLBCL of the elderly group showed OS (median OS: 20% as EBER cut-off: 17.0 vs. 32.2 months, *P* = 0.8434; 50% as EBER cut-off: 37.0 vs. 36.5 months, *P* = 0.8058) (Fig. 4a,c) and PFS (median PFS: 20% as EBER cut-off: 9.8 vs. 16.3 months, *P* = 0.5878; 50% as EBER cut-off: 20.7 vs. 20.5 months, *P* = 0.8323) similar to those of their young counterparts (Fig. 4b,d). In addition, we also analyzed the survival differences with other age cut-offs (40 or 60 years old), however, no difference of OS or PFS was recognized between different age groups with EBV positive DLBCL (data not shown).

## Discussion

While using 20% and 50% as cut-off vaues, the incidences of EBV positive DLBCL were 14.0% (35/250) and 10.4% (26/250), respectively. Our result was similar to that of a previous study in Peru (14.9% for 20% cut-off and 9.0% for 50% cut-off)^26^. However, taking different cut-off vaues used in previous studies into consideration, the incidence of EBV positive lymphoma appeared to be higher than those reported in previous studies in Asian countries^4^,^20^.

In current study, EBV positivity, defined by either EBER cut-off (20% or 50%), had a close association with male sex, advanced clinical stage, poor PS status and lower response to first-line treatment, which was observed in both young and elderly patients. These results indicated that EBV positive DLBCL were clinically aggressive, irrespective of age. Based on these, we then analyzed the association between EBV positivity and the clinical prognostic indicators reported previously^27^,^28^,^29^,^30^,^31^,^32^. The results showed that EBV positivity had a strong relationship with elevated serum CRP level, β2M level, CA125 level, ESR level and ferritin level, regardless of age and EBER cut-off value.

We subsequently included CD10, Bcl6, MUM1, FOXP1, GCET1 and LMO2 in our study as these markers were used to establish a diagnosis and further classify DLCBL into GCB or non-GCB subtype^21^,^22^,^23^,^24^. Research showed the majority of EBV positive DLBCL of the elderly had a non-GCB predominance, which is a subtype with poor prognosis. In our study, we accessed the COO with four conventional IHC algorithms. Although EBV positive DLBCL of the elderly group showed less frequent FOXP1 expression and the young group demonstrated less common of LMO2 expression, the EBV positive DLBCL (both age groups), although not statistically significant, demonstrated the non-GCB subtype preference than negative ones. However, the COO difference was observed in Choi and Visco-Young algorithms alone. The poor concordance among IHC algorithms may be one of reasons to explain our results, since 20–30% cases among the IHC algorithms are discrepant^22^,^33^. We also analyzed CD5, Myc, Bcl2, Ki-67 expression and DPE, MYC and BCL2 gene rearrangement and DHL because of their efficacy in prognostication^34^,^35^,^36^. We also chose CD30 due its significant association with EBV positivity in DLBCL that was observed in several previous studies^7^,^15^,^37^. Moreover, EBER^+^/CD30^+^ DLBCL had significantly poorer outcomes compared with EBER^−^/CD30^+^ cases^38^. Using the cut-off values of these proteins applied in above studies^7^,^34^,^35^,^36^,^37^, we accessed the incidences of these pathological factors in DLBCL according to EBV status and age. Interestingly, EBER positivity was associated with less frequent Bcl6 expression, which was also confirmed by previous studies^7^. Moreover, Constitutive expression of EBV-derived miRNAs, including BART3, BART7, BART9 and BART17-5p, was found to be capable of repressing Bcl6 expression, partly accounting for reduced Bcl6 expression in EBV positive lymphoma^39^. The proportions of Myc expression, DPE, MYC rearrangement and DHL were much higher in EBV positive DLBCL, which were consistent with the aggressive biological behavior of EBV positive DLBCL^1^,^11^,^13^. DPE and DHL were more frequent in EBV positive cases than EBV negative ones, which was inconsistent with the study by Ok *et al.*^7^, probably due to different ethnic background or geographic variation of EBV strains. Bcl2 expression was less frequent in EBV positive DLBCL. However, this was not observed in a previous study^7^. Of note, in that study, cut-off for Bcl2 was 70%, rather than 50%. Higher Ki-67 expression was observed in EBV positive patients, suggesting that EBV infection contributes the aggressiveness of EBV positive DLBCL^40^. Similar to previous studies^40^, the significant association of CD30 with EBV positivity in DLBCL was also confirmed in our study. However, the mechanism underlying this phenomenon remains to be determined. It is possible that CD30 functions synergistically with EBV to transform B lymphocytes^41^.

In present study, although the treatment for EBV positive DLBCL was more intensive than negative ones (much higher percentage of immunochemotherapy in EBV positive DLBCL), consistent with most studies^4^,^12^,^16^,^19^, both age groups with EBV positive DLBCL showed significantly worse OS and PFS than negative cases. Further comparison showed that EBV positive DLBCL of the elderly group had similar OS and PFS to the young counterparts. It is worth mentioning that the EBV positive DLBCL patients (both elderly and young) were treated with similar regimens. Actually, EBV positive DLBCL of the elderly was included as a provisional entity in the 2008 WHO classification of tumors of hematopoietic and lymphoid tissues, in which the WHO Working Group considered that there was insufficient evidence to recognize this entity as a distinct disease at that time^3^,^42^. EBV positive DLBCL of the young group that had no evidence of underlying immunosuppression had been described in previous reports^4^,^16^. The identification of these young cases arised an interrogation that if EBV positive DLBCL might be an entity that was not restricted to patients who were older than 50 years old alone. However, one recent study did not recognized unfavorable baseline characteristics in young patients with EBV positive DLBCL^14^. Therefore, they concluded that EBV positive DLBCL of young group should be considered to be a distinct clinical entity different from EBV positive DLBCL of the elderly^14^. In present study, to say the least, no distinct difference was recognized in the clinical and pathological features between EBV positive DLBCL in the elderly and the young group, which was in accordance with most studies^4^,^12^. These unfavorable factors were shared by both groups, which indicated that EBV positive DLBCL belongs to a unique entity with aggressive clinical features^7^,^11^,^13^,^14^,^15^,^20^,^43^, regardless of age. Since there is no uniform cut-off for EBV positivity, some reports attributed the inconsistent results to the different cut-off values used in different studies^7^,^11^,^13^,^14^,^44^,^45^,^46^. However, by appling two most frequently-used cut-off values, our study indicated that EBER positive patients harbored more unfavorable clinical and pathological features than EBER negative ones, regardless of the cut-off values of EBV.

Several studies had reported that EBV positive patients showed an inferior prognosis compared with EBV negative cases, especially the elderly group^7^,^11^,^13^,^15^,^18^,^47^,^48^. The clinical course is often aggressive with a median survival of 2 years and an overall 5-year survival rate of approximately 25%^4^,^5^,^8^. In our study, accordantly, EBV positive DLBCL of the elderly showed worse survival than negative counterparts. Many studies also showed the young patients of EBV positivity demonstrated poor outcome with traditional immunochemotherapy^4^,^12^,^16^,^19^, which was confirmed in current study. However, the study by Hong *et al.* revealed young EBV positive DLBCL patients (6.7%, 13/195) had an outcome similar to EBV negative ones^14^.

In fact, the biological mechanism underlying similar clinical and pathological features and outcome between these age groups remains to be investigated. To elucidate molecular mechanisms involved EBV positive DLBCL, Ok *et al.*^7^ evaluated GEP profiles signatures of DLBCL with different EBV status. It is worth noting that in the 24 EBV positive patients included in that study, 7 patients were younger than 50 years old. They revealed the NF-κB activation and JAK/STAT activation were enhanced in EBV positive DLBCL compared with negative counterparts by gene set enrichment analysis. Thus, EBV positive DLBCL might share similar GEP signatures and common pathogenic pathways, irrespective of age. It also has been reported that ABC-like DLBCL more frequently exhibited JAK-STAT and NF-κB pathways activation compared with GCB-like DLBCL^49^,^50^ Interestingly, Kato *et al.* demonstrated that NF-κB and JAK-STAT pathways were more remarkably activated in EBV positive DLBCL compared with ABC-like EBV negative DLBCL^47^. Using miRNA array platforms, Andrade *et al.*^43^ showed that miR-146b and miR-222 were highly specific for EBV positive DLBCL. The targets of hsa-miR-146b and its viral counterpart are INTS6 and IPO, both of which are tumor suppressors^51^ and mediators of inflammation^26^ Similarly, hsa-miR-222 interferes with important proteins related to oncogenesis, cell cycle regulation, cell transcription, cell adhesion, oxidative stress, and apoptosis inhibition^52^. Besides, NF-κB pathway can be activated indirectly by hsa-miR-222^43^. All of the above findings suggested that EBV positive DLBCL shared a common molecular basis, irrespective of age.

Additionally, using other age cut-off values, we did not recognized any significant differences of survival between elderly and the young patients of EBV positivity. This also indicated that the young group patients should not be excluded from the whole cohort of EBV positive patients and the 50 years old cut-off might not be applicable in the R-CHOP era.

In summary, EBV positive DLBCL patients shared poor prognostic features, regardless of elderly or young group. The survival analysis also showed that EBV DLBCL of the elderly showed a similar outcome to the young ones. Based on these results, we suggest that the age criterion, and possibly the name-EBV positive DLBCL of elderly itself, be modified in future.

## Additional Information

**How to cite this article**: Lu, T.-X. *et al.* Epstein-Barr virus positive diffuse large B-cell lymphoma predict poor outcome, regardless of the age. *Sci. Rep.* **5**, 12168; doi: 10.1038/srep12168 (2015).

## Figures and Tables

**Table 1:** Patients’ characteristics in different groups (EBER cut-off: 20%).

Variable	Number.(%)	**All patients (N** **=** **250)**		*P* value	**Elderly group**		*P* value	**Young group**		*P* value
		**EBV+**	**EBV−**		**EBV+**	**EBV−**		**EBV+**	**EBV−**	
		**Number. (%)**			**Number. (%)**			**Number. (%)**		
Age	250	35	215		25	141		10	74	
≥50y	166 (66.4)	25 (71.4)	141 (65.6)	0.497	25	141	NA	NA	NA	NA
<50y	84 (33.6)	10 (28.6)	74 (34.4)		NA	NA		10	74	
Sex	250	35	215		25	141		10	74	
Male	144 (57.6)	27 (77.1)	117 (54.4)	**0.012**	19 (76.0)	76 (53.9)	**0.040**	8 (80.0)	41 (55.4)	0.182
Female	106 (42.4)	8 (22.9)	98 (45.6)		6 (24.0)	65 (46.1)		2 (20.0)	33 (45.6)	
Site	250	35	215		25	141		10	74	
Extranodal	106 (41.6)	20 (57.1)	86 (40.0)	0.057	15 (60.0)	62 (44.0)	0.139	5 (50.0)	24 (32.4)	0.303
Lymph node	144 (58.4)	15 (42.9)	129 (60.0)		10 (40.0)	79 (56.0)		5 (50.0)	50 (67.6)	
Stage	250	35	215		25	141		10	74	
I/II	125 (50.0)	12 (34.3)	113 (52.6)	**0.045**	8 (32.0)	68 (48.2)	0.133	4 (40.0)	45 (60.8)	0.307
III/IV	125 (50.0)	23 (65.7)	102 (47.4)		17 (68.0)	73 (51.8)		6 (60.0)	29 (39.2)	
ECOG PS	238	25	213		15	144		10	69	
0–1	207 (83.5)	16 (64.0)	191 (89.7)	**0.002**	9 (60.0)	126 (87.5)	**0.013**	7 (70.0)	65 (94.2)	**0.040**
2–4	31 (16.5)	9 (36.0)	22 (10.3)		6 (40.0)	18 (12.5)		3 (30.0)	4 (5.8)	
ESI	242	30	212		20	139		10	73	
0–1	194 (80.2)	20 (66.7)	174 (82.1)	**0.048**	13 (65.0)	112 (80.6)	0.143	7 (70.0)	62 (84.9)	0.361
>1	48 (19.8)	10 (33.3)	38 (17.9)		7 (35.0)	27 (19.4)		3 (30.0)	11 (15.1)	
IPI	237	29	208		19	139		10	69	
0–2	174 (73.4)	17 (58.6)	157 (75.5)	0.054	9 (47.4)	93 (66.9)	0.095	8 (80.0)	64 (92.8)	0.214
3–5	63 (26.6)	12 (41.4)	51 (24.5)		10 (52.6)	46 (33.1)		2 (20.0)	5 (7.2)	
Treatment	250	35	215		25	141		10	74	
R-CHOP	125 (50.0)	27 (77.1)	98 (45.6)	NA	19 (76.0)	58 (41.1)	NA	8 (80.0)	40 (54.1)	NA
R-DA- EPOCH	35 (14.0)	7 (20.0)	28 (13.0)	NA	5 (20.0)	23 (16.3)	NA	2 (20.0)	5 (6.8)	NA
CHOP	90 (36.0)	1 (2.9)	89 (41.4)	NA	1 (4.0)	60 (42.6)	NA	0 (0.0)	29 (39.1)	NA
Response	250	35	215		25	141		10	74	
CR(u)/PR	212 (84.8)	23 (65.7)	189 (87.9)	**0.001**	16 (64.0)	119 (84.4)	**0.025**	7 (70.0)	70 (94.6)	**0.034**
No response	38 (15.2)	12 (34.3)	26 (12.1)		9 (36.0)	22 (15.6)		3 (30.0)	4 (5.4)	

Abbreviations: EBV: Epstein-Barr virus; ESI: extranodal sites involvement; ECOG PS: Eastern Cooperative Oncology Group Performance Status; IPI: International Prognostic Index; NCCN: National Comprehensive Cancer Network; Chemo: R-CHOP, rituximab, cyclophosphamide, doxorubicin, vincristine, and prednisone; R-DA-EPOCH: Dose-adjusted EPOCH-R ([etoposide, prednisone, vincristine, cyclophosphamide, doxorubicin] + rituximab); CHOP, cyclophosphamide, doxorubicin, vincristine, and prednisone; chemotherapy; CR(u): complete remission(unconfirmed); PR: partial remission.

**Table 2:** Patients’ characteristics of in different groups (EBER cut-off: 50%).

Variable	Number.(%)	**All patients (N** **=** **250)**		*P* value	**Elderly group**		*P* value	**Young group**		*P* value
		**EBV+**	**EBV−**		**EBV+**	**EBV−**		**EBV+**	**EBV−**	
		**Number. (%)**			**Number. (%)**			**Number. (%)**		
Age	250	26	224		19	147		7	77	
>50y	166 (66.4)	19 (73.1)	147 (65.6)		19 (100)	147 (65.6)	NA	NA	NA	NA
≤50y	84 (33.6)	7 (26.9)	77 (34.4)		NA	NA		7 (100)	77 (34.4)	
Sex	250	26	224		19	147		7	77	
Male	144 (57.6)	21 (80.8)	123 (54.9)	**0.012**	15 (78.9)	80 (54.4)	**0.042**	6 (85.7)	43 (55.8)	0.230
Female	106 (42.4)	5 (19.2)	101 (45.1)		4 (21.1)	67 (45.6)		1 (14.3)	34 (44.2)	
Site	250	26	224		19	147		7	77	
Extranodal	106 (41.6)	11 (42.3)	95 (42.4)	0.992	9 (47.4)	68 (46.3)	0.927	2 (28.6)	27 (35.1)	1.000
Lymph node	144 (58.4)	15 (57.7)	129 (57.6)		10 (52.6)	79 (53.7)		5 (71.4)	50 (64.9)	
Stage	250	26	224		19	147		7	77	
I/II	125 (50.0)	6 (23.1)	119 (53.1)	**0.004**	4 (21.1)	72 (49.0)	**0.021**	2 (28.6)	47 (61.0)	0.122
III/IV	125 (50.0)	20 (76.9)	105 (46.9)		15 (78.9)	75 (51.0)		5 (71.4)	30 (39.0)	
ECOG PS	238	20	218		13	146		7	72	
0–1	207 (83.5)	14 (70.0)	193 (88.5)	**0.001**	9 (69.2)	126 (86.3)	0.111	5 (71.4)	67 (93.1)	0.115
2–4	31 (16.5)	6 (30.0)	25 (11.5)		4 (30.8)	20 (13.7)		2 (28.6)	5 (6.9)	
ESI	242	21	221		14	145		7	76	
0–1	194 (80.2)	16 (76.2)	178 (80.5)	0.577	10 (71.4)	115 (79.3)	0.501	6 (85.7)	63 (82.9)	1.000
>1	48 (19.8)	5 (23.8)	43 (19.5)		4 (28.6)	30 (20.7)		1 (14.3)	13 (17.1)	
IPI	237	20	217		13	145		7	72	
0–2	174 (73.4)	11 (55.0)	163 (75.1)	0.051	6 (46.2)	96 (66.2)	0.224	5 (71.4)	67 (93.1)	0.115
3–5	63 (26.6)	9 (45.0)	54 (24.9)		7 (53.8)	49 (33.8)		2 (28.6)	5 (6.9)	
Treatment	250	26	224		19	147		7	77	
R-CHOP	125 (50.0)	23 (88.5)	102 (45.6)	NA	18 (94.7)	59 (40.1)	NA	5 (71.4)	43 (55.8)	NA
R-DA- EPOCH	35 (14.0)	2 (7.6)	33 (14.7)	NA	0 (0.0)	28 (19.1)	NA	2 (28.6)	5 (6.5)	NA
CHOP	90 (36.0)	1 (2.9)	89 (39.7)	NA	1 (5.3)	60 (40.8)	NA	0 (0.0)	29 (37.7)	NA
Response	250	26	224		19	147		7	77	
CR(u)/PR	212 (84.8)	18 (69.2)	194 (86.6)	**0.037**	13 (68.4)	135 (91.8)	**0.008**	5 (71.4)	73 (94.8)	0.076
No response	38 (15.2)	8 (30.8)	30 (13.4)		6 (31.6)	12 (8.2)		2 (28.6)	4 (5.2)	

Abbreviations: EBV: Epstein-Barr virus; ESI: extranodal sites involvement; ECOG PS: Eastern Cooperative Oncology Group Performance Status; IPI: International Prognostic Index; NCCN: National Comprehensive Cancer Network; Chemo: R-CHOP, rituximab, cyclophosphamide, doxorubicin, vincristine, and prednisone; R-DA-EPOCH: Dose-adjusted EPOCH-R ([etoposide, prednisone, vincristine, cyclophosphamide, doxorubicin] + rituximab); CHOP, cyclophosphamide, doxorubicin, vincristine, and prednisone; chemotherapy; CR(u): complete remission(unconfirmed); PR: partial remission.

**Table 3:** Clinical features of patients in different groups (EBER cut-off: 20%).

Variable	Number.(%)	**All age**		*P* value	**Elderly group**		*P* value	**Young group**		*P* value
		**EBV+**	**EBV−**		**EBV+**	**EBV−**		**EBV+**	**EBV−**	
		**Number. (%)**			**Number. (%)**			**Number. (%)**		
CRP	117	20	97		14	62		6	35	
Over ULN	57 (48.7)	15 (75.0)	42 (43.3)	**0.010**	10 (71.4)	28 (45.2)	0.076	5 (83.3)	14 (40.0)	0.080
Normal	60 (51.3)	5 (25.0)	55 (56.7)		4 (28.6)	34 (54.8)		1 (16.7)	21 (60.0)	
β2MG	134	22	109		14	72		8	40	
Over ULN	59 (44.0)	16 ()	43 (72.7)	**0.003**	10 (71.4)	33 (45.8)	0.080	6 (75.0)	10 (25.0)	**0.012**
Normal	75 (56.0)	6 ()	69 (27.3)		4 (28.6)	39 (54.2)		2 (25.0)	30 (75.0)	
TK1	92	15	77		9	52		6	25	
Over ULN	28 (30.4)	4 (26.7)	24 (31.2)	1.000	2 (22.2)	15 (28.8)	1.000	2 (33.3)	9 (36.0)	1.000
Normal	64 (69.6)	11 (73.3)	53 (68.8)		7 (77.8)	37 (71.2)		4 (66.7)	16 (64.0)	
CA125	108	18	90		13	58		5	32	
Over ULN	42 (38.9)	13 (72.2)	29 (32.2)	**0.001**	8 (61.5)	17 (29.3)	0.051	5 (100.0)	12 (37.5)	**0.014**
Normal	66 (61.1)	5 (27.8)	61 (67.8)		5 (38.5)	41 (70.7)		0 (0.0)	20 (62.5)	
ESR	163	22	141		14	88		8	53	
Over ULN	49 (30.1)	19 (86.4)	30 (21.3)	**<0.001**	13 (92.9)	20 (22.7)	**<0.001**	6 (75.0)	10 (18.9)	**0.003**
Normal	114 (69.9)	3 (13.6)	111 (78.7)		1 (7.1)	68 (77.3)		2 (25.0)	43 (81.1)	
Ferritin	152	24	128		18	82		6	46	
Over ULN	52 (34.2)	15 (62.5)	37 (28.9)	**0.001**	10 (55.6)	24 (29.3)	**0.033**	5 (83.3)	13 (28.3)	**0.015**
Normal	100 (65.8)	9 (37.5)	91 (71.1)		8 (44.4)	58 (70.7)		1 (16.7)	33 (71.7)	
LDH	250	34	216		24	139		10	77	
Over ULN	114 (45.6)	20 (58.8)	94 (43.5)	0.096	15 (62.5)	59 (42.4)	0.068	5 (50.0)	35 (45.5)	1.000
Normal	136 (54.4)	14 (41.2)	122 (56.5)		9 (37.5)	80 (57.6)		5 (50.0)	42 (54.5)	
CK	149	24	125		16	70		8	55	
Complex	5 (3.4)	1 (4.2)	4 (3.2)	0.590	1 (6.2)	2 (2.9)	0.465	0 (0.0)	2 (3.6)	1.000
Normal	144 (96.6)	23 (95.8)	121 (96.8)		15 (93.8)	68 (97.1)		8 (100.0)	53 (96.4)	
BM	150	25	125		17	81		8	44	
Positive	17 (11.3)	4 (16.0)	13 (10.4)	0.487	3 (17.6)	8 (9.9)	0.398	1 (12.5)	5 (11.4)	1.000
Negative	133 (88.7)	21 (84.0)	112 (89.6)		14 (82.4)	73 (90.1)		7 (87.5)	39 (88.6)	

Abbreviations: EBV: Epstein-Barr virus; CRP: serum C reactive protein; ULN: upper limit of normal; β2MG: β2 microglobulin; ESR: erythrocyte sedimentation rate; LDH: lactate dehydrogenase; CK: chromosome karyotype; BM: bone marrow.

**Table 4:** Clinical features of patients in different groups (EBER cut-off: 50%).

**Variable**	**Number.(%)**	**All age**		***P* value**	**Elderly group**		***P*** **value**	**Young group**		***P*** **value**
		**EBV+**	**EBV-**		**EBV+**	**EBV-**		**EBV+**	**EBV-**	
		**Number. (%)**			**Number. (%)**			**Number. (%)**		
CRP	117	19	98		13	63		6	35	
Over ULN	57 (48.7)	15 (78.9)	42 (42.9)	**0.004**	10 (76.9)	28 (44.4)	**0.033**	5 (83.3)	14 (66.7)	0.080
Normal	60 (51.3)	4 (21.1)	56 (57.1)		3 (23.1)	35 (55.6)		1 (16.7)	21 (33.3)	
β2MG	134	18	116		11	75		7	41	
Over ULN	59 (44.0)	14 (77.8)	45 (38.8)	**0.002**	9 (81.8)	34 (45.3)	**0.024**	5 (71.4)	11 (26.8)	**0.033**
Normal	75 (56.0)	4 (22.2)	71 (61.2)		2 (18.2)	41 (54.7)		2 (28.6)	30 (73.2)	
TK1	92	14	78		8	53		6	25	
Over ULN	28 (30.4)	4 (28.6)	24 (30.8)	1.000	2 (25.0)	15 (28.3)	1.000	2 (33.3)	9 (36.0)	1.000
Normal	64 (69.6)	10 (71.4)	54 (69.2)		6 (75.0)	38 (71.7)		4 (66.7)	16 (64.0)	
CA125	108	16	92		11	60		5	32	
Over ULN	42 ()	12 (75.0)	30 (32.6)	**0.001**	7 (63.6)	18 (30.0)	**0.043**	5 (100.0)	12 (37.5)	**0.014**
Normal	66 ()	4 (25.0)	62 (67.4)		4 (36.4)	42 (70.0)		0 (0.0)	20 (62.5)	
ESR	163	21	142		14	88		7	54	
Over ULN	49 (30.1)	18 (85.7)	31 (21.8)	**<0.001**	13 (92.9)	20 (22.7)	**<0.001**	5 (71.4)	11 (20.4)	**0.011**
Normal	114 (69.9)	3 (14.3)	111 (78.2)		1 (7.1)	68 (77.3)		2 (28.6)	43 (79.6)	
Ferritin	152	21	131		16	84		5	47	
Over ULN	52 (34.2)	14 (66.7)	38 (29.0)	**0.001**	10 (62.5)	24 (28.6)	**0.009**	4 (80.0)	14 (29.8)	**0.043**
Normal	100 (65.8)	7 (33.3)	93 (71.0)		6 (37.5)	60 (71.4)		1 (20.0)	33 (70.2)	
LDH	250	25	225		18	145		7	80	
Over ULN	114 (45.6)	14 (56.0)	100 (44.4)	0.271	10 (55.5)	64 (44.1)	0.359	4 (57.1)	36 (45.0)	0.698
Normal	136 (54.4)	11 (44.0)	125 (55.6)		8 (45.5)	81 (55.9)		3 (42.9)	44 (55.0)	
CK	149	21	128		14	72		7	56	
Complex	5 (3.4)	1 (4.8)	4 (3.1)	0.537	1 (7.1)	2 (27.8)	0.417	0 (0.0)	2 (3.6)	1.000
Normal	144 (96.6)	20 (95.2)	124 (96.9)		13 (92.9)	70 (97.2)		7 (100.0)	54 (96.4)	
BM	150	22	128		15	83		7	45	
Positive	17 (11.3)	4 (18.2)	13 (10.2)	0.279	3 (20.0)	8 (9.6)	0.366	1 (14.3)	5 (11.1)	1.000
Negative	133 (88.7)	18 (81.8)	115 (89.8)		12 (80.0)	75 (90.4)		6 (85.7)	40 (88.9)	

Abbreviations: EBV: Epstein-Barr virus; CRP: serum C reactive protein; ULN: upper limit of normal; β2MG: β2 microglobulin; ESR: erythrocyte sedimentation rate; LDH: lactate dehydrogenase; CK: chromosome karyotype; BM: bone marrow.

**Table 5:** Pathological features of patients in different groups (EBER cut-off: 20%).

**Variable**	Number.(%)	**All age**		***P*** **value**	**Elderly group**		***P*** **value**	**Young group**		***P*** **value**
		**EBV+**	**EBV−**		**EBV+**	**EBV−**		**EBV+**	**EBV−**	
		**Number.(%)**			**Number.(%)**			**Number.(%)**		
CD10	250	35	215		25	141		10	74	
Positive	60 (24.0)	4 (11.4)	56 (26.0)	0.060	4 (16.0)	35 (24.8)	0.338	0 (0.0)	21 (28.4)	0.060
Negative	190 (76.0)	31 (88.6)	159 (74.0)		21 (84.0)	106 (75.2)		10 (100)	53 (71.6)	
Bcl6	248	35	223		25	140		10	73	
Positive	160 (64.5)	12 (34.3)	148 (66.4)	**<0.001**	7 (28.0)	91 (65.0)	**0.001**	5 (50.0)	57 (78.1)	0.113
Negative	88 (35.5)	23 (65.7)	65 (33.6)		18 (72.0)	49 (35.0)		5 (50.0)	16 (21.9)	
MUM1	250	35	215		25	141		10	74	
Positive	157 (62.8)	23 (65.7)	134 (62.3)	0.700	16 (64.0)	95 (67.4)	0.741	7 (70.0)	39 (52.7)	0.500
Negative	93 (37.2)	12 (34.3)	81 (37.7)		9 (36.0)	46 (32.6)		3 (30.0)	35 (47.3)	
GCET1	245	32	213		22	141		10	72	
≥60%	31 (12.7)	3 (9.4)	28 (13.1)	0.776	2 (9.1)	18 (12.8)	1.000	1 (10.0)	10 (13.9)	1.000
<60%	214 (87.3)	29 (90.6)	185 (86.9)		20 (90.9)	123 (87.2)		9 (90.0)	62 (86.1)	
FOXP1	230	32	198		22	130		10	68	
≥60%	138 (60.1)	17 (53.1)	121 (61.1)	0.392	10 (45.5)	89 (68.5)	**0.036**	7 (70.0)	32 (47.1)	0.176
<60%	92 (35.9)	15 (46.9)	77 (38.9)		12 (54.5)	41 (31.5)		3 (30.0)	36 (52.9)	
LMO2	244	32	222		22	140		10	72	
≥30%	194 (79.5)	25 (78.1)	169 (76.1)	0.439	19 (86.4)	107 (76.4)	0.412	6 (60.0)	62 (86.1)	0.062
<30%	50 (20.5)	7 (21.9)	33 (23.9)		3 (13.6)	33 (23.6)		4 (40.0)	10 (13.9)	
CD5	250	35	215		25	141		10	74	
Positive	17 (6.8)	2 (5.7)	15 (7.0)	1.000	1 (4.0)	7 (5.0)	1.000	1 (10.0)	8 (10.8)	1.000
Negative	233 (93.2)	33 (94.3)	200 (93.0)		24 (96.0)	134 (95.0)		9 (90.0)	66 (89.2)	
CD30	250	35	215		25	141		10	74	
Positive	25 (10.0)	10 (28.6)	15 (6.5)	**0.001**	7 (28.0)	9 (6.4)	**0.003**	3 (30.0)	6 (6.8)	**0.070**
Negative	225 (90.0)	25 (71.4)	200 (93.4)		18 (72.0)	132 (93.6)		7 (70.0)	68 (93.2)	
Myc	248	33	215		23	141		10	74	
Positive	91 (36.7)	19 (57.6)	72 (33.5)	**0.008**	14 (60.9)	53 (37.6)	**0.035**	5 (50.0)	19 (25.7)	0.140
Negative	157 (63.3)	14 (42.4)	143 (66.5)		9 (39.1)	88 (62.4)		5 (50.0)	55 (74.3)	
Bcl2	249	35	214		25	140		10	74	
Positive	142 (57.0)	14 (40.0)	128 (59.8)	**0.028**	10 (40.0)	87 (62.1)	**0.038**	4 (40.0)	41 (55.4)	0.503
Negative	107 (43.0)	21 (60.0)	86 (40.2)		15 (60.0)	53 (37.9)		6 (60.0)	33 (44.6)	
DPE	64 (25.6)	15 (42.9)	49 (22.8)	**0.012**	11 (44.0)	35 (24.8)	**0.048**	4 (40.0)	14 (18.9)	0.210
Others	186 (74.4)	20 (57.1)	166 (77.2)		14 (56.0)	106 (75.2)		6 (60.0)	60 (81.1)	
Ki-67	249	34	215		24	141		10	74	
≥70%	97 (39.0)	23 (67.6)	74 (34.4)	**<0.001**	17 (70.8)	48 (34.0)	**0.001**	6 (60.0)	26 (35.1)	0.170
<70%	152 (61.0)	11 (23.4)	141 (65.6)		7 (29.2)	93 (66.0)		4 (40.0)	48 (64.9)	
MYC-ba	246	32	214		23	140		9	74	
Positive	33 (13.4)	10 (31.2)	23 (10.7)	**0.004**	7 (34.4)	15 (10.7)	**0.018**	3 (33.3)	8 (10.8)	0.094
Negative	213 (86.6)	22 (68.8)	191 (89.3)		16 (69.6)	125 (89.3)		6 (66.7)	66 (89.2)	
BCL2/IGH	247	32	215		23	141		9	74	
Positive	32 (13.0)	7 (21.9)	25 (11.6)	0.152	4 (17.4)	19 (13.5)	0.535	3 (33.3)	6 (8.1)	0.054
Negative	215 (87.0)	25 (78.1)	190 (88.4)		19 (82.6)	122 (86.5)		6 (66.7)	68 (91.9)	
DHL	6 (2.4)	4 (12.1)	2 (1.0)	**0.003**	3 (13.0)	1 (1.0)	**0.009**	1 (10.0)	1 (1.4)	0.225
Non-DHL	242 (97.6)	29 (87.9)	213 (99.0)		20 (87.0)	140 (99.0)		9 (90.0)	73 (98.6)	
Hans	250	35	215		25	141		10	74	
GCB	98 (39.2)	14 (40.0)	84 (39.1)	0.917	9 (36.0)	49 (34.8)	0.904	5 (50.0)	35 (47.3)	1.000
Non-GCB	152 (60.8)	21 (60.0)	131 (60.9)		16 (64.0)	92 (65.2)		5 (50.0)	39 (52.7)	
Choi	244	23	221		22	140		10	72	
GCB	110 (45.1)	10 (43.5)	100 (45.2)	0.092	6 (27.3)	59 (42.1)	0.186	4 (40.0)	41 (56.9)	0.335
Non-GCB	134 (54.9)	22 (56.5)	112 (54.8)		16 (72.7)	81 (57.9)		6 (60.0)	31 (43.1)	
Tally	246	32	214		22	141		10	73	
GCB	68 (27.6)	11 (34.4)	57 (26.6)	0.361	7 (31.8)	35 (24.8)	0.485	4 (40.0)	22 (30.1)	0.717
Non-GCB	178 (72.4)	21 (65.6)	157 (73.4)		15 (68.2)	106 (75.2)		6 (60.0)	51 (69.9)	
Visco-Young	246	34	212		24	140		10	72	
GCB	106 (43.1)	11 (32.4)	95 (44.8)	0.173	6 (25.0)	57 (40.7)	0.144	5 (50.0)	38 (52.8)	1.000
Non-GCB	140 (56.9)	23 (67.6)	117 (55.2)		18 (75.0)	83 (59.3)		5 (50.0)	34 (47.2)	

Abbreviations: EBV: Epstein-Barr virus; DPE: double protein expression; ba: break apart; DHL: double hit lymphoma; GCB:germinal center B cell.

**Table 6:** Pathological features of patients in different groups (EBER cut-off: 50%).

Variable	Number.(%)	**All age**		***P*** **value**	**Elderly group**		***P*** **value**	**Young group**		***P*** **value**
		**EBV+**	**EBV−**		**EBV+**	**EBV−**		**EBV+**	**EBV−**	
		**Number.(%)**			**Number.(%)**			**Number.(%)**		
CD10	250	26	224		19	147		7	77	
Positive	60 (24.0)	3 (11.5)	57 (25.4)	0.116	3 (18.6)	36 (24.5)	0.568	0 (0.0)	21 (27.3)	0.184
Negative	190 (76.0)	23 (88.5)	167 (74.6)		16 (81.2)	111 (75.5)		7 (100.0)	56 (72.7)	
Bcl6	248	26	222		19	146		7	76	
Positive	160 (64.5)	6 (23.1)	154 (69.4)	**<0.001**	4 (21.1)	94 (64.4)	**<0.001**	2 (28.6)	60 (78.9)	**0.010**
Negative	88 (35.5)	20 (76.9)	68 (30.6)		15 (78.9)	52 (35.6)		5 (71.4)	16 (21.1)	
MUM1	250	26	224		19	147		7	77	
Positive	157 (62.8)	15 (57.7)	142 (63.4)	0.569	11 (57.9)	100 (68.0)	0.377	4 (57.1)	42 (54.5)	1.000
Negative	93 (37.2)	11 (42.3)	82 (36.6)		8 (42.1)	47 (32.0)		3 (42.9)	35 (45.5)	
GCET1	245	23	222		16	147		7	75	
≥60%	31 (12.7)	1 (4.3)	30 (13.5)	0.326	0 (0.0)	20 (13.6)	0.223	1 (14.3)	10 (13.3)	1.000
<60%	214 (87.3)	22 (95.7)	192 (86.5)		16 (100.0)	127 (86.4)		6 (85.7)	65 (86.7)	
FOXP1	230	23	207		16	136		7	71	
≥60%	138 (60.1)	10 (43.5)	128 (61.8)	0.088	6 (37.5)	93 (68.4)	**0.014**	4 (57.1)	35 (49.3)	1.000
<60%	92 (35.9)	13 (56.5)	79 (38.2)		10 (62.5)	43 (31.6)		3 (42.9)	36 (50.7)	
LMO2	244	23	221		16	146		7	75	
≥30%	194 (79.5)	17 (73.9)	177 (80.1)	0.586	14 (87.5)	112 (76.7)	0.527	3 (42.9)	65 (86.7)	**0.014**
<30%	50 (20.5)	6 (26.1)	44 (19.9)		2 (12.5)	34 (26.3)		4 (57.1)	10 (13.3)	
CD5	250	26	224		19	147		7	77	
Positive	17 (6.8)	2 (7.7)	15 (6.7)	1.000	1 (5.3)	7 (4.8)	1.000	1 (14.3)	8 (10.4)	1.000
Negative	233 (93.2)	24 (92.3)	209 (93.3)		18 (94.7)	140 (95.2)		6 (85.7)	69 (89.6)	
CD30	250	26	224		19	147		7	77	
Positive	25 (10%)	10 (38.5)	15 (6.7)	**<0.001**	7 (36.8)	9 (6.1)	**<0.001**	3 (42.9)	6 (7.8)	**0.024**
Negative	225 (90.0)	16 (61.5)	209 (93.3)		12 (63.2)	138 (93.9)		4 (57.1)	71 (92.2)	
Myc	248	24	224		17	147		7	77	
Positive	91 (36.7)	12 (50.0)	79 (35.3)	0.155	9 (52.9)	58 (39.5)	0.284	3 (42.9)	21 (27.3)	0.402
Negative	157 (63.3)	12 (50.0)	145 (64.7)		8 (47.1)	89 (60.5)		4 (57.1)	56 (72.7)	
Bcl2	249	26	223		19	146		7	77	
Positive	142 (57.0)	8 (30.8)	134 (60.1)	**0.004**	6 (31.6)	91 (62.3)	**0.010**	2 (28.6)	43 (55.8)	0.242
Negative	107 (43.0)	18 (69.2)	89 (39.9)		13 (8.4)	55 (37.7)		5 (71.4)	34 (44.2)	
DPE	64 (25.6)	8 (30.8)	56 (25.0)	0.523	6 (31.6)	40 (27.2)	0.689	2 (28.6)	16 (20.8)	0.639
Others	186 (74.4)	18 (69.2)	168 (75.0)		13 (68.4)	107 (72.8)		5 (71.4)	61 (79.2)	
Ki-67	249	25	224		18	147		7	77	
≥70%	97 (39.0)	17 (68.0)	80 (35.7)	**<0.001**	12 (66.7)	53 (36.1)	**0.001**	5 (71.4)	27 (35.1)	0.100
<70%	152 (61.0)	8 (32.0)	144 (64.3)		6 (33.3)	94 (63.9)		2 (28.6)	50 (64.9)	
MYC-ba	246	23	223		17	146		6	77	
Positive	33 (13.4)	6 (26.1)	27 (12.1)	0.099	4 (23.5)	18 (12.3)	0.252	2 (33.3)	9 (11.7)	0.178
Negative	213 (86.6)	17 (73.9)	196 (87.9)		13 (76.5)	128 (87.7)		4 (66.7)	68 (88.3)	
BCL2/IGH	247	23	224		17	146		6	77	
Positive	32 (13.0)	6 (26.1)	26 (11.6)	0.170	3 (17.6)	20 (13.7)	0.711	3 (50.0)	6 (7.8)	**0.015**
Negative	215 (87.0)	17 (73.9)	198 (88.4)		14 (82.4)	127 (86.3)		3 (50.0)	71 (92.2)	
DHL	6 (2.4)	3 (12.5)	3 (1.3)	**0.013**	2 (11.8)	2 (1.4)	0.054	1 (14.3)	1 (1.3)	0.161
Non-DHL	242 (97.6)	21 (87.5)	221 (98.7)		15 (88.2)	145 (98.6)		6 (85.7)	76 (98.7)	
Hans	250	26	224		19	147		7	77	
GCB	98 (39.2)	10 (38.5)	88 (39.3)	0.139	8 (42.1)	50 (34.0)	0.486	2 (28.6)	38 (49.4)	0.437
Non-GCB	152 (60.8)	16 (61.5)	136 (60.7)		11 (57.9)	97 (66.0)		5 (71.4)	39 (50.6)	
Choi	244	23	221		16	146		7	75	
GCB	110 (45.1)	6 (26.1)	104 (47.1)	0.054	5 (31.2)	60 (41.1)	0.446	1 (14.3)	44 (58.6)	**0.042**
Non-GCB	134 (54.9)	17 (73.9)	117 (52.9)		11 (68.8)	86 (58.9)		6 (85.7)	31 (41.3)	
Tally	246	23	223		16	147		7	76	
GCB	68 (27.6)	7 (30.4)	61 (27.4)	0.422	6 (37.5)	36 (24.5)	0.105	1 (14.3)	25 (32.9)	0.425
Non-GCB	178 (72.4)	16 (69.6)	162 (72.6)		10 (62.5)	111 (75.5)		6 (85.7)	51 (67.1)	
Visco-Young	246	25	221		18	146		7	75	
GCB	106 (43.1)	7 (28.0)	99 (44.8)	0.108	5 (27.8)	58 (39.7)	0.325	2 (28.6)	41 (54.7)	0.249
Non-GCB	140 (56.9)	18 (72.0)	122 (55.2)		13 (72.2)	88 (60.3)		5 (71.4)	34 (45.3)	

Abbreviations: EBV: Epstein-Barr virus; DPE: double protein expression; ba: break apart; DHL: double hit lymphoma; GCB:germinal center B cell.
